# That was just your life: rethinking dementia for advance directives

**DOI:** 10.3389/fpsyt.2024.1435560

**Published:** 2024-07-29

**Authors:** Franlu Vulliermet, Daan Kenis

**Affiliations:** Centre for Ethics, Department of Philosophy, University of Antwerp, Antwerp, Belgium

**Keywords:** dementia, Alzheimer’s disease, epistemic injustice, contributory injustice, advance directives, representations

## Abstract

Over the past decades, literature in dementia ethics has extensively looked at moral questions revolving around the care of older people living with dementia. Particularly prevalent are autonomy-related concerns regarding topics such as advance directives. In this paper, we argue that these discussions are crucially premised on how dementia is understood and represented. Despite the multiplicity of dementia presentations in people, the dominant discourse predominantly frames dementia as ‘monstrous,’ an ‘enemy,’ a destructive experience in need of eradication. We contend that such a monolithic approach, from a moral standpoint, is problematic in several respects. Indeed, framing heavily influences the way dementia is understood and experienced, leading to stigmatization, bias, and distress. Not only does it influence decisions and discussions on advance directives, but we argue that this flawed understanding of dementia is rooted in and contributes to epistemic harm. In the first section, we introduce the ethics of advance directives. More specifically, we introduce the view developed by Dworkin who has largely influenced the debate by making the case for advance directives by grounding them in the principles of autonomy and beneficence. In the second section, we show how dementia is still mostly framed monolithically as a ‘destructive experience.’ We then show that this framing is problematic because it oversteps the different pathologies dementia implies, which leads to an inaccurate representation of the condition. In the third section, we present possible alternative framings: dementia as normal aging, a person-centered care framework, and an embodied view. In the fourth section, drawing on recent developments in the epistemic injustice literature, we explore how maintaining and utilizing flawed understandings of dementia may lead to distinct moral-epistemic harms for those living with dementia and inform ongoing discussions on advance directives. Finally, in the concluding section, we return to the case of advance directives and what the implications of rethinking dementia are.

## Introduction

In *Still Alice* (1), Alice (portrayed by Julianne Moore), a linguistics professor, is diagnosed with early-onset familial Alzheimer’s disease. Aware of her declining state, Alice battles to delay the effects of dementia as much as possible. Nevertheless, as her decline becomes ineluctable, she formulates advance directives in the form of a recorded video addressed to her ‘future self,’ instructing her to take sleeping pills with a dosage that would implicitly lead to her death. Later in the movie, Alice stumbles upon the video her ‘then self’ had recorded without any recollection of its whole meaning or consequences. While she is following the instructions, she is interrupted by the arrival of her caregiver. Unable to remember what she was doing, Alice never takes the pills. The spectator may be appalled by Alice being unable to fulfill her wish to take her own life after reaching a stage her ‘then self’ had deemed unbearable. Nevertheless, a second reading conflicts with this interpretation, and the spectator should maybe rather rejoice that the attempt to take the pills failed. When she discovers the recording, Alice just had a video call with her younger daughter to help her rehearse a play while cooking simultaneously. Her ‘now self’ seems quite happy with her life and still engages in meaningful activities and exchanges. Looking again, one cannot then help but wonder if ‘then Alice’ would not have committed an irreparable wrong to ‘now Alice’ by instructing her to take her own life.

Dementia – as a clinical syndrome present in a variety of medical conditions and pathologies with distinct etiologies, such as Alzheimer’s disease (AD) – describes the decline in cognitive abilities to perform everyday activities (2). From a public health perspective, this decline, increasing with age, coupled with an extended life expectancy, affects a growing number of people. More colloquially, dementia is sometimes referred to as a ‘silver tsunami’ (3, 4). The challenges of dementia are numerous: while it has become a growing concern from a medical perspective, the rise in dementia cases equally presents economic, political, and ethical challenges (5, 6).

Over the past decades, literature in dementia ethics has extensively looked at moral questions revolving around the care of older people living with dementia. Particularly prevalent are autonomy-related concerns regarding topics such as advance directives or managing feeding needs (7, 8). As we argue throughout this article, these discussions are crucially premised on how dementia is understood and represented. Despite the multiplicity of dementia presentations in people, the dominant discourse predominantly frames dementia as ‘monstrous,’ an ‘enemy,’ a destructive experience in need of eradication (4).

In this paper, we contend that while such a monolithic approach may be relevant from a curative perspective, from a moral standpoint, this framing is problematic in several respects. Indeed, such framing heavily influences the way dementia is understood *and* experienced, leading to stigmatization, bias, and distress. Not only does it influence decisions and discussions on advance directives, but this flawed understanding of dementia may also be a source of epistemic harm. We proceed as follows: in the first section, we introduce the ethics of advance directives. More specifically, we introduce the view developed by Dworkin who has largely influenced the debate by making the case for advance directives grounding them in the principles of autonomy and beneficence. In the second section, we come back to how dementia is still mostly framed monolithically as a ‘destructive experience.’ We then show that this framing is problematic because it oversteps the different pathologies dementia implies, which leads to an inaccurate representation of the condition. In the third section, we present possible alternative framings: dementia as normal aging, a person-centered care framework, and an embodied view. In the fourth section, drawing on recent developments in the epistemic injustice literature, we explore how maintaining and utilizing flawed understandings of dementia may lead to distinct moral-epistemic harms for those living with dementia and inform ongoing discussions on advance directives. Finally, in the concluding section, we return to the case of advance directives and address the implications of rethinking dementia.

## The ethics of advance directives

The topic of dementia provides for a number of challenging moral quandaries and has, as such, been of primary concern to ethicists and legal scholars. Particularly pervasive are discussions on ‘advance directives.’ Advance directives are (generally) written statements stipulating instructions and preferences on future medical care in case of (expected) loss of decisional capacity (9). As a (legal) tool for surrogate decision-making, advance directives allow people to stipulate their wishes for future medical decisions. In general, advance directives are used as guidance for medical decision-making in the event the person is not able to express her will due to incapacitating circumstances such as cognitive impairments, being in a coma, after an accident, or because of illness.^1^ Since dementia involves (at least) a partial loss of the cognitive capacities deemed necessary to exercise autonomous decision-making, people draft advance directives to stipulate the conditions and procedure for surrogate decision-makers. Advance directives are then conceived as a means to extend a person’s autonomy to a ‘future self’ lacking this capacity.

Nevertheless, an important moral quandary arises when people with dementia express interests that run counter to their previously stipulated directives. If a person, prior to diagnosis, drafted a directive stipulating, for example, a ‘do not resuscitate’-order but later does express a wish to receive treatment, it is unclear to healthcare workers and her relatives which wishes should be honored. The debate in dementia ethics, then, primarily concerns the moral authority of advance directives and has mostly been framed around the concept of autonomy.

One of the most influential stances^2^ in this debate comes from Ronald Dworkin, who suggests that an advance directive reflects the individual’s judgment of her own life and should, therefore, be viewed as morally authoritative. Dworkin, in what is now seen as the received view (9), offers two arguments for this stance: the argument from autonomy and the argument from beneficence. With regards to the first, Dworkins defense of the moral authority of advance directives is premised on a conception of autonomy grounded in the integrity of the ‘then self,’ which Dworkin stipulates as “people’s general capacity to lead their lives out of a distinctive sense of their own character, a sense of what is important to and for them” (11, p. 224). Following Dworkin, people with dementia have lost this narrative capacity as they cannot reflect on their past or future (12). Therefore, in such cases, respecting the autonomy of the person entails respecting the preferences of the person before suffering from dementia, even over her current preferences. In practice, this makes an attractive case for advance directives, which evidently traces back to the (over)emphasized idea of rational autonomy present in Western bioethics (7) by allowing a person to take decisions over her ‘future self.’ This view culminated in the common (Western) bioethical assumption that extending a person’s autonomy using advance directives was ‘in principle correct’ (12). This approach is morally correct for many since the ‘then self’ in full possession of its faculties has a higher moral status.

Nevertheless, even if autonomy is taken to be of prime importance in dementia care, the principles of non-maleficence and beneficence state that physicians should not inflict unnecessary harm^3^ on patients and ensure their well-being, raising critical tensions regarding the effectuation of advance directives to stop treatment.^4^ Indeed, even if people with dementia are found to lack the capacity to act upon their interests, caregivers and proxy decision-makers should still act out of their best interests. To mitigate these concerns, Dworkin introduces a distinction between experiential and critical interests (11). Whereas the former is comprised of the quality of our own experiences and mostly constitutes pleasure, the latter involves thicker evaluations of who we are and what we hold to be important. Since critical interests are fundamental as opposed to temporary, Dworkin considers only critical interests as essential to our well-being. Since, according to Dworkin, dementia introduces a decisive break in one’s narrative self and therefore excludes the ability to have a temporally extended sense of self, people with dementia cannot engage with prior nor hold critical interests. Since the interests these people have are then ‘merely’ experiential – and when they conflict with the interests motivating the drafting of their advance directives – they fail to attain the moral weight that prior critical interests did.

Note, however, that the received view at several points relies on specific assumptions about what dementia is and how it is experienced. As Walsh suggests, the argument for autonomy “relies on us believing, like Dworkin does, that people with dementia in the moderate-late stage of the disease lack the mental capacities necessary to lead a life out of character” (9, p. 6). Indeed, we need to assume that the preferences people with dementia do evince, lack the stability and weight of those interests expressed prior to diagnosis or disease progression. Walsh continues that the argument from beneficence similarly relies on (i) the importance of ‘critical’ over other interests and (ii) the status of ‘critical interests’ being necessarily more stable and valid than any (relevant) interest expressed by the ‘now self.’

Moreover, the communicative practice of assessing the interests and capacities of the person with dementia by family members, caregivers, and others may itself be liable to misunderstanding. As we will explore at length later, such misunderstandings open the door to specific moral-epistemic harms. As the literature on epistemic injustice – or the wrong done to someone in their capacity as a knower – informs us, assessing the reliability of a speaker does not occur in a vacuum but is influenced by structural factors such as pervasive stereotypes and the concepts we have available to make sense of specific experiences. Given the centrality of assessments of interests and capabilities, the enactments of advance directives can then be said to crucially rely on our societal understanding of dementia or, more precisely, on how its representation affects our understanding of dementia and our engagement with those living with dementia. Similar concerns apply to the drafting of an advance directive. Indeed, when one decides to draft an advance directive in view of the expected loss of capacities one deems vitally important, one relies on several assumptions of the disease trajectory, outcomes, and, more generally, what it is like to live with dementia. Here, too, people rely on dominant understandings of dementia as they exist in our social imaginary.

As such, both the philosophical discussions on the moral authority of advance directives, the enactment of advance directives in care contexts, and the individual decision to draft an advance directive critically depend on one’s prior evaluation of the condition, which itself is deeply influenced by the dominant social representation of dementia. In the next section, we briefly sketch the dominant framing of dementia and suggest it relies on an impoverished clinical view of dementia, runs counter to the experiences of those living with dementia, and rests on a somewhat problematic philosophical understanding of identity.

## Framing dementia

As we underlined in the introduction, the way dementia has been (predominantly) framed unilaterally emphasizes a negative valence; dementia is deemed a ‘monster’ to eradicate. The dominance of this particularly horrid understanding of dementia, overshadowing the multiplicity of expression encapsulated within the syndrome dementia, has resulted in its monolithic framing in most discourses. While dementia describes cognitive decline with a wide variety of pathologies affecting people in numerous ways, in popular representations, dementia often (exclusively) takes the face of its most severe instantiations. This monolithic framing is notably characterized by the predominance of AD in dementia discourses, which has been established both as a paragon and a vernacular term for dementia through the lobbying^5^ of medical researchers and carers (13). This use has sedimented AD (and dementia) in the collective mind (or social imaginary) both amidst important segments of professional^6^ and lay audiences so strongly and negatively that it has resulted in the ‘idea’ of dementia being one of the most terrifying illnesses (15).

The widely-held perception of dementia as ‘terrifying’ is, in important ways, related to the perceived threats it poses to an individual’s capacities to exercise autonomy – a value critical to Western thinking and central to bioethics. Immanuel Kant, grounding morality in reason, contributed to the development of the Western notion of autonomy with the idea that being able to exercise full rational capacities would grant a higher moral status (7). Consequently, to be a ‘full person’ (in Western cultures) is linked to functions of rationality, memory,^7^ and the autonomy that goes with them (16). While not universally lauded, autonomy has become (one of) the main principles in bioethics (17).^8^ For instance, in some Asian countries, physicians do not fully disclose the medical condition to a patient when they believe it may harm them. There, the principle of beneficence trumps the one of autonomy. As dementia potentially deprives a person of her functions grounding autonomy, the issue has become core to dementia ethics.^9^ As references to people with dementia as being ‘mere vegetables’ (18) or living a ‘cabbage-like existence’ (3) evince, people suffering from dementia are often taken to have lost partly or totally their autonomy.

The use of such terms, while explicitly undermining, is consistent with the idea that people with dementia are deprived of the functions that make them ‘full persons.’ This is anchored in the current framing of dementia and is the expression of a certain form of reductionism where a person would equate to her brain, which, when it does not work correctly, a person would be a lesser human. These discourses express a stark contrast between the person before and after suffering from dementia. Beyond autonomy, people with dementia can also show changes in their behaviors and personality, reemphasizing how ‘terrifying’ dementia can be by also robbing a person of her memories. In other words, reflecting upon dementia involves thinking about practical and philosophic problems linked to identity.

Philosophically, identity can be interpreted in two different ways: either as numerical or as psychological (narrative) (19). Dementia does not affect numerical identity; the person is still ‘the same,’ there is a continuation of a single body going through changes. It does, however, alter psychological identity (19). This identity refers to the conception a person has of herself, who she is, who she should be, and who she wants to be. This second understanding, prevalent in sociology and psychology, takes a person to be capable of having several identities throughout her life when she changes jobs, marries, etc. (19). This is the problem with dementia in this perspective: people may do things they do not remember, do not recognize people they were close to before, or have radical changes in their personalities. Here, the framing of dementia comes back into play, with strong and explicit formulations and metaphors saying that the person is ‘gone,’ for instance, inevitably emphasizing the destructive experience dementia is. After all, the first AD patient, Auguste Deter herself, would repeatedly say, “*I have lost myself*” (16). Knowing if a person with dementia is ‘the same’ from a psychological perspective is a thorny question. A dominant conception, developed by Locke and after him by Dawkins, grounds identity in psychological continuity. A person is ‘the same’ only insofar as she has conscious remembering, that she can recall her previous states and accredit them to herself (19). Simply said, memory is therefore crucial for this conception of identity, and losing memory when suffering from dementia is tantamount to losing identity.^10^ Gerontological and dementia literature have then distinguished between the ‘then self’ that existed before the pathology and the ‘now self’ that lives in the present, with no or little recollection of the ‘then self’ (12).

### Issues with this framing of dementia

In this section, we highlight some of the issues with the dominating framing of dementia. First, the monolithic framing oversteps the different pathologies dementia implies, which leads to an inaccurate (medical) representation of the condition. This results in a misleading portrayal of dementia with (potentially) significant consequences. Notably, it overlooks that people may experience dementia in different ways, not only from their personal perspective or their social condition but also from a strictly biomedical point of view. Disease onset and progression vary widely across persons. Some lose the capacity to speak and forget words (aphasia), while others forget most of their memories. The multiplicity of the clinical image of dementia, then, implies that the (clinical) needs of two persons with dementia can be radically different. Identifying the form of dementia then is crucial: not only may it suggest that the person’s clinical needs will be different, but it also has clinical implications as rates of progression and prognosis are going to vary (22). This being said, recently and increasingly, even in the well-defined diagnostic category of AD, evidence suggests the need to recognize heterogeneity and the need to stratify people with dementia according to fine-grained disease characteristics. Nevertheless, further research and progress are still necessary here. Despite the need for a more granular biomedical view, Whitehouse himself still thinks in terms of a ‘wide range of persons who have often ‘similar needs’ (our emphasis) regardless of specific diagnosis’ (22). Indeed, while more granular diagnostic categories may function to improve dementia care and treatment, the issues identified transcend the clinical context and are, therefore, unlikely to be resolved within the biomedical purview.

Second, framing dementia solely as a ‘destructive’ experience is problematic because the ways people refer to dementia through words, stories, or discourses influence the way it is understood and experienced (4). This has implications from the perspective of the person receiving the diagnosis. Smedinga et al. (4) report that in lay contexts, a diagnosis of AD is often taken to amount to demolishing a person’s life, ‘bringing sadness and despair.’ Unsurprisingly, as Post (1993) observed, such framing has sparked international debate over physician-assisted suicide as people increasingly started considering it as an option after receiving a diagnosis of AD. It also marked an increase in setting up advance directives (4). Furthermore, this framing also shapes how others and society treat people with dementia, notably how to communicate with them (23 More importantly, the framing of dementia also participates in the conception we have of the ones who suffer from it, leading to a moral stance with practical and ethical implications for how we treat them (3). As we showed previously, the framing allows for discourses undermining these people by comparing them to vegetables and being incapacitated.

Thirdly, even the ethical literature expounds on this ‘defective” aspect, encouraging distinguishing between the person before dementia and after, especially to justify the relevance of advance directives.^11^ We contend that this leads to another issue with how this particular framing is operationalized in the context of advance directives, namely that the distinction between the ‘then-now’ self is misleading. For one, as we saw, if we take identity in its numerical understanding, there is and will always be one person. Furthermore, even severely demented people retain some continuity between the ‘now’ and ‘then self.’ Even when such continuity may seem to be totally gone, it may simply be ‘dormant.’ Aquilina and Hughes recount the story of Mrs. G., who suffered from dementia and was mute and not interacting with her husband. After taking an anti-dementia drug, she showed tremendous improvements. Her case demonstrates that even when the self seems to have disintegrated, it actually may persist (15). The case of Mrs. G, which is not unique, leads us to believe that something of the ‘self’ remains even if dementia brings significant changes to a person’s identity.

The monolithic framing of dementia is, therefore, problematic in several respects. First, it is erroneous within the frame of biomedicine since, as we have stated, dementia is a syndrome encompassing different conditions. Hence, it may lead to a misunderstanding of what dementia is among lay audiences. Notably, the fact that it does not affect everyone in a single unified way means that there are actually many unknowns in the prognosis (22). As we have shown, such thoughts are mistaken; they lack the granularity necessary to understand the variety of conditions dementia brings together, and it overlooks that predicting the exact extent of the cognitive decline after diagnosis is currently not possible. It is all the more problematic because misleading people has practical consequences starting already with the diagnosis, which itself can amplify the disability that could result from the pathology (3). This leads to the second aspect: the framing by being misleading may result in mistreatment and harming of people with dementia. Picturing an inevitable cognitive worsening akin to annihilating the person contributes to stigma and harming people with dementia (4). We need to highlight that what makes the framing especially problematic here is that the wrong done to them is insidious and pervasive. Pervasive because it is widespread and unavoidable: most stories, discourses, or diagnoses put an emphasis on the destructive aspects of the pathology. Insidious because this emphasis may lead to the viewing and defining of people with dementia primarily in defective terms, resulting in a malignant positioning leading relatives and carers to behave disrespectfully albeit unwittingly (24). Far from being an epiphenomenon, professional literature and lay public press is rife with malignant positioning.^12^ Following Smedinga et al. (4), we advance (and will explore further) that current framings and jargon may be harmful and ought to be carefully used when communicating to lay audiences, media, or elsewhere. Because framings can steer people’s understanding and be linked to moral appeals (4), it is a powerful tool to use, and reframing dementia can help us better understand and treat people suffering from it.

### Reframing dementia

While, as stated previously, the negative framing of dementia is pervasive across lay and professional contexts, discursive spaces explicitly reframing dementia and offering important counternarratives exist.^13^ For one, scholarship in dementia studies in dialogue with and supported by activist organizations such as the Young Dementia Network and DEEP have engaged in uprooting pervasive issues with problematic dementia narratives and advocated for different understandings of it (25). In this section, we highlight some of the ways in which reframing dementia would be possible. In the introduction, we characterized dementia as present in a broad range of pathologies and medical conditions characterized by a decline in cognitive abilities. The prospects of cognitive decline turn dementia into an often terrifying diagnosis. For many, losing personality, identity, or memories may register as a condition as fearsome as death (12). While we do not intend to underestimate the potentially severe implications of cognitive decline, we want to suggest that beyond these destructive aspects, cognitive decline is part of life and can be framed differently.

One alternative framing is to consider cognitive decline (and dementia) as part of ‘normal aging.’ Most will experience some form of cognitive decline over their lives, whether or not that decline meets the diagnostic thresholds of dementia. Researchers have suggested that the differences between age-associated decline in cognitive functioning and dementia are more quantitative than qualitative (26). Moreover, it has been suggested that distinguishing between ‘normal,’ age-related cognitive decline and ‘cognitive decline’ resulting from a neurocognitive condition at a neurobiological level is difficult (27). That is a reason why defining AD’s boundaries precisely, for instance, is still complex because all of its individual features occur in normal aging to some extent (28). With aging, we generally become forgetful; people with dementia – according to this view – are ‘just’ more forgetful. Even if this position was recently reformulated, considering the memory difficulties and behavioral changes coming from dementia as related to normal aging is not new. This view was dominant in Western cultures until the 1970s (26). In fact, ‘dementia-as-normal-aging’ was once considered a fruitful explanatory model to understand dementia. However, it has since been partially abandoned due to its problematic implications for therapeutic contexts. In what could be called a ‘social model of dementia,’ stigma and suffering are explained mainly in reference to ageist social conditions. In societies where older people were respected and revered, people with dementia held similar esteem, whereas, in ageist societies, they suffered from dementia and were treated like other older people (although they arguably suffered more as they were more vulnerable and had less coping capacities) (26). A strictly social model of dementia, however, has difficulty recognizing the vulnerability and specific needs associated with the pathophysiology of dementia. Moreover, in terms of care, no special treatments or additional health resources were expected to be given to people with dementia over and above the ones for older people (26). Additionally, within a social model of dementia, it is difficult to account for harms (e.g., dizziness, sexual dysfunction, blood pressure, etc.) directly associated with dementia pathophysiology. For such reasons, this explanatory model was progressively abandoned and replaced by others, notably dementia as a neuropsychiatric condition that, despite its disparate etiology, is the result of underlying progressive brain disease. The framing we have been criticizing so far is grounded in this latter model of dementia, which is still dominant among professionals and lay audiences.

Nevertheless, this model has already been heavily criticized in the past for neglecting social and psychological factors. Moreover, for some, it was deemed too reductionist in its biological determinism and could not account for different facts about the social reality of dementia (26). Kitwood, notably, had significantly contributed to a change of perspectives advocating that ‘the person comes first’ (29). He proposed a new explanatory model by introducing the use of person-centered care (PCC) to distinguish a certain type of care approach from more medical and behavioral approaches to dementia (30). In this model, dementia is considered a dialectical interplay between neurological and social-psychological factors (31). Emphasizing the latter allows for a more comprehensive and less deterministic understanding of people with dementia. His view flourished and was impactful, notably through its influence on the biopsychosocial model of illness the WHO promoted (26).

As explanatory models have moved away from ‘normal aging’ to be more comprehensive and put the emphasis on the person and her needs to provide appropriate care, it may seem awry and counterintuitive to advocate for it. Nonetheless, we contend that this explanatory model still has value and can foster a better understanding of people with dementia. First, holding on to one explanatory model does not preclude excluding the others. Individuals or societies can hold several simultaneously or fluctuate between them (26). For this reason, we do not believe that a normal aging explanatory model necessarily entails not giving special treatments and care to people with dementia compared to other older people. On the contrary, we think that seeing dementia as inscribed in the process of normal aging can and ought to be compatible with models such as the one of Kitwood that highlights the need for interpersonal care aiming at the preservation and enhancement of the personhood (29, 31). With this mindset, the value of resorting to ‘normal aging’ lies in its potential to break down the barriers between ‘us’ and ‘them.’ Gubrium (32) argued that the attempts to establish distinctions at a neurobiological level were rather a social construction from the cognitive functioning ones to set them apart. Post (33) was going in the same direction when he observed a persistent bias against people with cognitive disabilities. If we want to include people with dementia and care for them, it requires us to deconstruct these barriers we have erected individually and collectively (23) and that a misleading framing of dementia perpetuates. Seeing dementia and the declines that go along with it as ‘normal’ rather than as ‘defective and destructive’ can emphasize our common humanity. Having this commonality in mind would allow us to relate to people with dementia in most respects with the same considerate and caring ways we relate to others (23).

While the PCC model represented a breakthrough in understanding and caring for people with dementia (amongst other conditions), substantial criticism has since emerged, emphasizing PCC’s shortcomings, especially from the perspective of nursing staff and caregivers (34). In short, these critiques underline that neither Kitwood nor his contemporaries properly considered the resources and implications for caring staff and the structural changes required to treat people with dementia according to PCC principles (34). Although their concerns do not question the benefits of PCC, (Critical) Dementia Studies have moved to another stage, beyond a merely medical or social model of disability, as they engage in the shared project of ‘de-centering’ or revising notions of self and personhood and their associations with forms of power by grounding them in concepts such as relationality or embodiment (34). Embodiment in dementia, while maintaining personhood, eludes the reductionist account where a person would equate to her brain by looking at how dementia is expressed bodily and not strictly in cognitive ways. Fuchs, for instance, advances a conception of personhood rooted in the phenomenology of the body: selfhood is primarily vital and bodily (16). In short, for Fuchs, everything we do, consciously or not, has a bodily foundation that is never totally lost, even in the case of dementia. He justifies it by grounding the continuity of a person in body memory, the experiences sedimented in the body through life rather than in the repertoire of memories stored in the brain (16). Without expounding further on these views, resorting to concepts of embodied personhood can change how we view and treat people with dementia.

Rather than framing dementia on exclusively cognitivist accounts supporting views of autonomy, a relational embodied account stresses the importance and relevance of viewing people with dementia in their environmental and social contexts, in their own individual embodiment (16). Hence, although dementia remains a destructive experience, as without question, it deprives people of capacities such as reflective thought, which are crucial for one’s own sense of identity, embodied approaches such as the one put forward by Fuchs emphasize that habits, sensory, and mentor memories remain, nonetheless. Even if Deter was saying ‘she lost her-self,’ she had to retain some sense of self to be able to state it, highlighting again that the ‘self’ was not simply totally gone and lost. So, while it does not discount destructive features of dementia, understanding selfhood as essentially bodily, we can arrive at a different perception of people with dementia: not just people who have lost their rationality and would be less than persons, but on the contrary as persons with bodily and intercorporeal personhood realized as long as they keep living in appropriate surroundings (16). Furthermore, adopting such a view and stressing the importance of the environment for the person with dementia allows us to advocate for the necessity to reconsider her and what appropriate biomedical care would be. In short, it comes down to rethinking whether it is the care networks that are not adapted rather than viewing the person with dementia as alienated (35).^14^ (Body) memory is increasingly taken into consideration to understand dementia, with the purview to revise notions of personhood. More specifically, critical dementia studies have emphasized the need to rethink the ‘category of people with dementia’ to understand better the lived experience of these people (25, p. 5). We want to stress the critical importance of following this way, supporting initiatives and opportunities such as the one of Sandberg and Ward, who have encouraged people with dementia to write about their life experiences and openly share their perspectives using different (creative) forms such as photo reports.

## Epistemic injustice and the framing of dementia

As stipulated previously, the predominance of a specific, negatively-laden monolithic framing of dementia may have significant implications regarding social stigma and the treatment of people with dementia. This seems particularly problematic since alternative resources rooted in dementia experience and subsequent academic engagement with those experiences suggest different, productive ways for treating (people with) dementia. In this section, we diagnose this tension and suggest it plays an important part in perpetuating the dominance of a reductionistic framing of dementia, which itself fosters distinct epistemic and practical harms. More explicitly, we contend that this unilateral, reductionist understanding of dementia is rooted in and propagates various forms of *epistemic* injustice. After a short introduction to Miranda Frickers’ initial account of epistemic injustice, we suggest that the uniliteral framing of dementia is perpetuated by an active and persistent ignorance per the biomedical community.

It is precisely this contributory injustice that lies at the root of and exacerbates the testimonial and hermeneutical injustice people with dementia are vulnerable to.

### Epistemic injustice

Miranda Fricker coined ‘epistemic injustice’ to stipulate the harm resulting from “(…) a wrong done to someone specifically in their capacity as a knower” (36, p.1). She argues that being wronged as an epistemic subject is to be wronged in a capacity essential to human value (36, p. 44). In addition to the primary harm of objectification, failing to express one’s epistemic agency often involves particular practical harms as well. In the context of dementia, we will show how epistemic injustice may lead to communicative issues impacting dementia care, as well as implicate discussions on advance directives.

Fricker distinguishes between two forms of epistemic injustice. Testimonial injustice concerns the prejudicial deflation of a speaker’s credibility based on an identity-related stereotype. The prevalent dismissal of women’s testimony on the grounds of it being overly emotional and subjective constitutes exactly the type of prejudicial credibility deficit Fricker captures in testimonial injustice. In addition to prejudicial credibility deflation, unjustified *in*flation of credibility can similarly result in testimonial injustice. Since credibility is a comparative good, the attribution of credibility to one person may result in a proportionate downgrade of another’s credibility. An overestimation of an actor’s epistemic authority can then result in a related testimonial injustice (37, 38). Hermeneutical injustice, the second form of epistemic injustice, occurs when an epistemic subject is hampered in understanding or communicating their experiences due to a hermeneutical gap in our collective repository of epistemic resources^15^ owing to the structural exclusion of particular identities from meaning-making practices. Fricker provides the example of Carmita Wood, who, before the widespread availability and uptake of the term ‘sexual harassment,’ experienced distinct moral and practical harms due to an inability to understand and communicate her experiences of (workplace) sexual misconduct.

Subsequent literature has expanded significantly on Fricker’s initial account to include a variety of other ways in which the epistemic agency – i.e., the ability to use, contribute to, and transform knowledge of subjects – can be thwarted (40). Drawing on this literature, in what follows, we argue that insistence on the conceptual framing of dementia as strictly detrimental and destructive despite the availability of other (complementary or even superior) ways to conceptualize dementia is rooted in a form of *actively and structurally maintained ignorance* (41). That is to say that, despite the availability of alternative means to understand dementia, societal and medical discourse largely (and structurally) ignores alternative contributions to the dementia imaginary. The recalcitrance of this flawed framing of dementia may be morally problematic since (i) it is based on a wrongful epistemic exclusion and persistent failure to engage with the understandings that arise from communities of people with dementia, and (ii) results in significant moral-epistemic and practical harms through depriving prospective people suffering from dementia the means to properly understand dementia and rendering the experiences of dementia unintelligible further deflating their credibility as interlocutors. It is important to note from the outset, then, that the epistemic harms associated with dementia are not merely the result of vicious caregivers or healthcare professionals – or *bad apples* – but rather have important structural origins and, therefore, require structural solutions.

### Contributory injustice

As previously described, the framing of dementia, as it took hold in the social imaginary, seems to espouse a persistent yet unilateral and ultimately flawed understanding of the breadth of experiences of living with dementia, such as experiences of lucidity, adaptive interests, and expressions of personal growth. Moreover, despite the available alternative perspectives on dementia arising from people’s experiences, activist groups, and patient organizations and validated by academic engagements in aging and dementia studies, the representations we draw upon in public debate, biomedical discourse, and popular culture still seem oblivious to such counternarratives. This persistent failure to engage with what are arguably more informed, better representations of dementia owes to what Kristie Dotson has labeled ‘contributory injustice’ (42).

Contributory injustice entails a dominant epistemic agent’s or institution’s willful and situated ignorance “in maintaining and utilizing structurally prejudiced [epistemic] resources that result in epistemic harm to the epistemic agency of the knower” (42, p. 9). Contra Fricker’s treatment of ‘collective epistemic resources,’ Dotson suggests that marginalized groups often do develop an alternative set of epistemic resources that run counter to a dominant understanding. Indeed, in order to make sense of the specificities of experiences of oppression, typically not shared by dominant groups, marginalized knowers generally do or are even required to devise and share their own concepts, languages, and understandings. As such, while they, over time, collectively develop a linguistic sense of understanding of their experiences, a central issue to the perpetuation of epistemic injustices lies in that these resources often fail to garner uptake within dominant communities. We should, therefore, distinguish between *dominant* epistemic resources and *extant* resources arising in and through the experiences of marginalized people. It follows, then, that the issues related to hermeneutical injustice do not exist only in the unavailability but rather in a persistent neglect of these resources and the experiences they accompany in dominant knowers. Recall the example of ‘sexual harassment’ arising from shared workplace experiences of women. This concept first needed to find uptake beyond ‘consciousness-raising groups’ and, notably, with those in the position to affect (political) change (institutions, academic administrations, etc.) before the harms related to hermeneutical injustice could be mitigated. Despite the availability of epistemic resources sensitive to their experiences and oppression, marginalized groups are often impeded in *contributing* this knowledge to the conceptual repository operative within the relevant context, i.e., the set of dominant epistemic resources.

The aforementioned recalcitrance of a dominant view of dementia un- (or minimally) informed by more nuanced resources arising in patient, activist, and academic spaces patients is indicative of contributory injustice. While more nuanced resources do exist, are widely shared among dementia communities and patients, and find support in academic spaces and some healthcare professionals (see footnote 6), they generally fail to garner substantial uptake in broader societal dementia discourse. This is morally problematic for two reasons. First, it constitutes an injustice in itself since those in relevant meaning-making positions fail to show the necessary epistemic due diligence with regard to the resources available. Given that these alternative resources offer an important complementary understanding of the phenomenon of interest, i.e., dementia, and are reasonably available, the burden of proof with regards to their irrelevance falls with those staying with the monolithic understanding offered above. Second, and importantly, the epistemic exclusion of these resources (and these epistemic communities more generally) in relevant meaning-making practices (i.e., institutions, clinical practice, and academic philosophy) may have important downstream consequences on the experiences of those living with dementia and dementia care alike. For example, while more nuanced resources to understand living with dementia exist, the resistance they encounter when transitioning to the wider conceptual repertoire may block (prospective) people with dementia’s access to helpful, more nuanced tools to make sense of their own experiences. More generally, the unavailability of these – often more adequate – resources constitutes epistemic harm to the wider community to the extent that family members or caregivers are denied access to such tools, potentially hampering proper (self-)understanding (43). This might then contribute to, perpetuate, and even intensify hermeneutical and testimonial injustice.

### Hermeneutical injustice

Recall that hermeneutical injustice concerns the harms that occur through the unavailability of epistemic resources necessary to make sense of or communicate one’s social experiences owing to a structural exclusion from dominant meaning-making practices. As Kidd and Carel (2014), Kidd and Carel (44) have explored extensively, hermeneutical injustice is prevalent in clinical practice. Patients’ experiences are not generally sought out, considered, or even wholly excluded from policy and research (45). Indeed, although there are some improvements in engaging patient representatives and organizations in biomedical research, clinical boards, and policy-making, historically, patients have rarely been consulted or asked to participate in the development of clinical epistemic resources (44). This may, in turn, introduce several difficulties for self-understanding and communicating illness experiences.

For one, hermeneutical injustice may arise when the resources necessary to convey first-personal aspects of illness are not (sufficiently) available or acceptable in the clinical imaginary. Given that our extant epistemic resources on illness are primarily informed by a biomedical framework – focusing on biological dysfunction rather than illness experience – patients may encounter difficulties in conveying important social and phenomenological aspects of living with illness. Caregivers may, for example, fail to understand or see the significance of prevalent illness experiences such as feelings of loss, bodily betrayal, and social exclusion (44). Second, hermeneutical injustice may also occur through a lack of resources to understand *particular conditions*. People suffering from so-called contested conditions such as CFS/ME, fibromyalgia, and more recently Long Covid, or conditions that are unfamiliar to large swaths of healthcare professionals, such as endometriosis, often take years to arrive at a diagnosis (46–48).^16^ This, too, constitutes a hermeneutical injustice since the exclusion of these resources is in part attributable to a prior marginalization of these patients in medical meaning-making. The wider unavailability of those resources to understand patient conditions hampers attempts to communicate their experiences to and with healthcare professionals.

Both forms of hermeneutical injustice have been described in the context of dementia as well. Given that dominant narratives on dementia characterize this experience as dominated by loss, suffering, and decline, it is clear that those living with dementia are vulnerable to hermeneutical injustice to the extent that other, more nuanced resources are unavailable, hampering (self-)understanding and communication. For one, framing dementia in the ways explored above may directly inform the communication opportunities of people with dementia. As Kitwood reminds us, malignant social processes, resulting in and perpetuating the infantilization and disempowerment of people with dementia, may lead others to be unperceptive to – i.e., lack the necessary resources to understand – the meanings of people with dementia being conveyed, and hence, deny the person with dementia’s standing as a semiotic subject (Sabat and Harré, 1994). This may be particularly problematic for those living with dementia outside the frame of its societal representation. Dementia activist Helga Rohra recounts several instances of her and fellow activists’ dementia status being cast in doubt due to a limited understanding of what dementia can be (Rohra, 2023). For one, she recalls a physician questioning the structured speech in which a fellow activist expressed herself. During the presentation of her own book, *Stepping out of the Shadows*, an audience member interjected that, surely, she had to be an actor; someone with dementia would never be this articulate (Rohra, 2023). Similar issues of communication and understanding are rife in the literature. Snyder (23) relates the story of a person suffering from AD who, during a support group meeting, expresses concerns about having less authentic exchanges because others treat them (patients with AD) with a ‘you need help’ attitude. These examples evoke how a limited understanding on the part of medical professionals and lay audiences may impact both how those living with dementia are treated in healthcare and society more generally. Compare this to cases of young onset dementia, where patients experience significant delays in attaining diagnosis due to physicians taking their concerns less seriously since they present as ‘healthy’ or ‘still working’ or prodromal symptoms (such as sleep disorders, behavioral alterations, or motor symptoms) of dementia not being registered as such, but rather as psychiatric conditions (O’Malley et al., 2021). In these cases, rather than strict communicative difficulties, it is a limited view of the clinical presentation of dementia that hinders healthcare professionals in proper diagnosis. Finally, the dominant framing of dementia as a deleterious and destructive experience also discounts the possibility of understanding that dementia does not preclude positive appraisals of life and well-being. As Hertogh et al. emphasize, recently diagnosed AD patients tend to adapt to the new realities of their condition – resulting in pushing back or canceling earlier set advance directives. Akin to a ‘disability paradox,’ those living with dementia often transition towards a positive outlook on life (Hertogh et al., 2007).^17^ These and similar experiences can be adequately understood as downstream effects of contributory injustices. Since those in positions that affect our wider understanding of dementia fail to engage with extant resources arising from those with relevant illness experience, hermeneutical gaps in our dominant frameworks persist, resulting in issues of understanding and communication of dementia experiences. This, moreover, has further downstream effects on how people with dementia are perceived by their interlocutors. Indeed, as José Medina notes, hermeneutical injustices may function to produce ‘social forms of blindness and deafness’ that impact communicative practices as well (37). Indeed, contributory and subsequent hermeneutical injustice may disadvantage people with dementia in communication by rendering their experiences unintelligible or nonsensical, reinforcing their vulnerability to testimonial injustice.

### Testimonial injustice

Recall that testimonial injustice involves the prejudicial (preemptive or reactive) de- or inflation of a speaker’s credibility based on identity-related stereotypes (36, 37). People living with dementia may be particularly vulnerable to this species of epistemic injustice. We suggest three reasons why this may be the case.

First, considering testimonial injustice in the case of dementia, we can straightforwardly advance that the current linguistic, cultural, and biomedical representations of dementia operationalize a variety of negative stereotypes that may function to deflate people with dementia’s credibility, epistemic authority, and reliability. Constructions of dementia as ‘identity-consuming,’ ‘hopeless,’ and ‘total and irrevocable’ influence how people with dementia are perceived as persons but also as epistemic agents. This, however, is generally unfounded since these severe forms of cognitive dysfunction rarely arise, if only in severe cases of late-stage dementia (49). In addition to the total and global loss of cognitive reliability, Young and colleagues have identified a variety of stereotypes perpetuated in various contexts fueling defective ascriptions of people with dementia’s credibility. Several metaphors prevalent in portrayals of dementia carry distinct epistemic valences. Portrayals of dementia as ‘a return to childhood,’ a ‘mindless body,’ or patients as ‘empty shells’ reinforce an understanding of patients as effectively unreliable, naive, or defective interlocutors. Finally, the expectation and anticipation of future loss have also been identified as effectively deflating people with dementia’s credibility beyond reason (44, 50).

Second, as stipulated above, the dominant hermeneutical resources on which caregivers and others rely to understand dementia seem misaligned with the actual experiences of dementia. While this – as stated earlier – constitutes a potential hermeneutical injustice, it may also reinforce testimonial injustice. When patients rely on particular expressions or (non-propositional) ways of conveying experiences that seem to run counter to an established understanding of dementia, they might come across as nonsensical or confused, further deflating their credibility.

Thirdly, while dementia may, in general, increase one’s vulnerability to testimonial injustice, people with late-stage dementia may be particularly susceptible to credibility discounting. Dementia progression is often accompanied by language impairments such as issues with phonology, syntax, or vocabulary (49) or even a complete loss of linguistic abilities. This results in the reliance on non-verbal communication, including pointing, pulling towards, pushing away, and facial and artistic expressions to convey basic needs, interests, or demands in exchanges with family members, friends, or caregivers. While the (partial) loss of linguistic abilities may hamper communication, it does not, in principle, inhibit those dependent on non-verbal communication from expressing epistemic agency as proverbial ‘speakers.’

Spencer (49) argues that we do generally take gesture, movement, and other forms of non-verbal expressions as epistemically loaded - we do discern some sense of meaning from gestures and other bodily expressions. While a lack of linguistic capacity on the part of the ‘speaker,’ at face value, limits one’s epistemic agency, the ‘epistemic loadedness’ of non-verbal communication and our general sensibilities to assess meaning in gestures, movements, and facial expressions means we can, in principle, extend attributions of epistemic agency to non-verbal knowers as well. If this is generally so, a failure to (selectively) extend these sensibilities to (non-verbal) people living with dementia, denying the epistemic-loadedness of their expressions, and therefore depriving them of their epistemic agency constitutes an additional form of (non-verbal) testimonial injustice.

Drawing upon recent empirical evidence, Spencer suggests that people with late-stage dementia are rarely allowed to exercise their epistemic agency. Even in care contexts, the non-verbal testimonies of dementia people rarely register as epistemically-loaded. They can, therefore, be subjected to a specific non-verbal form of testimonial injustice. This can occur pre-emptively when a hearer *a priori* decides not to engage with the person given an expectation of their lack of communicative capabilities – effectively silencing the person. On the other hand, a caregiver may engage with the person but register their non-verbal expressions as meaningless, delusional, or not epistemically charged (49).

While these concerns primarily track credibility *deficits* impacting how those living with dementia are treated as knowers, credibility excesses (37) of other actors might similarly implicate how those living with dementia are treated in epistemic practices. Consider the following case based on personal experiences: Mrs. M, an 89-year-old widow, was living alone in her house and suffered from early symptoms of dementia. One day, after falling, her condition worsened. She had difficulties moving around, showed short-term amnesia, and had difficulties finding words to express herself. She had to go to a medical retirement home and was placed under judicial protection. Shortly after, a physician came to assess her state, declaring that Mrs. M could no longer express her will concerning her situation. As time passed, Mrs. M showed improvements, with good and bad days. On bad days, she would be exhausted, barely react to people around her, and seemed lost. On good days, she remembered a lot of her past life, recognized people, and could follow basic discussions. However, as the legal procedure followed its course, discussions, and decisions concerning Mrs. M were taken without consulting her based on the initial medical certificate issued by the physician. Rather than merely an instance of a prejudicial credibility deficit, the case of Mrs. M highlights the dynamics of excessive credibility attributions in dementia care. Although her relatives offered a more nuanced view of Mrs. M.’s cognitive state, and Mrs. M. herself was intermittently capable of expressing her will and interests, the (single) assessment of a healthcare professional (crystallized in a medical certificate) was granted authority over other testimonies – both in clinical and legal contexts. The point here is that those living with dementia are not merely liable to prejudicial ascriptions of a credibility deficit; their (and their relatives) testimony is often judged inferior to that of a clinician or other healthcare professional.

The analysis above suggests that those living with dementia may be particularly susceptible to a variety of epistemic injustices, increasing their vulnerabilities to moral-epistemic and practical harms (including improper care). Importantly, while these harms may arise due to epistemically vicious caregivers or interlocutors, our treatment of hermeneutical and contributory injustice suggests that these are better characterized as downstream consequences of structural issues in how dementia is framed and treated in dominant meaning-making practices. Crucial to the perpetuation of these harms is the persistence of a monolithic framing and resistance to marginalized resources in public debate, clinical practice, and popular culture. While these issues primarily affect dementia care, the operationalization of a dominant, impoverished understanding of dementia also informs both the practice of and discussions on advance directives. This will be our focus in the next section, where we argue that dementia ethics (i) has to contend with the pervasiveness of epistemic injustice in dementia and (ii) when continuing to draw on and forward a partial view of dementia, it risks perpetuating these injustices.

## Return to advance directives

As stated, the decline in cognitive abilities associated with dementia represents several challenges and ethical dilemmas where autonomy and beneficence are in the balance in the perspective of the interests of the person with dementia. Advance directives have emerged over time as a powerful tool allowing a person to have her wishes respected in the event she would not be able to formulate them later on. Despite this promising prospect, advance directives often complexify decisions rather than simplifying them when the will and desires of the ‘now self’ of the person suffering from dementia conflict with the ones of her ‘then self.’ While this has sparked discussions and debates, the overall tendency is to honor the moral authority of advance directives, even in such conflicting cases. The position we defend in this paper is that this received view on advance directives may be more problematic than it appears and requires additional scrutiny.

The main reason we put forward is that advance directives are rooted in a framing of dementia, which is itself highly contentious. The dominating framing of dementia has drawn a stark contrast between the person before and after suffering from dementia. Discourses and representations conveyed amidst both clinical and lay audiences have cemented the idea that dementia constitutes a dramatic and destructive experience, a ‘monster’ that leaves nothing of the person you once were behind. Portrayals of dementia in popular culture, like in movies such as *Still Alice* or, more recently, *The Father*, end with their main characters totally lost and debilitated, as if they were mere shadows of their former self, and have also fostered the crystallization of the idea than the ‘now self’ is nothing in comparison to the ‘then self.’ Advance directives essentially appeal to the principle of autonomy, which is deeply rooted in Western bioethics and pervasive in this framing of dementia. As such, we have to highlight that resorting to them in this context and holding to them with high value makes sense because they are the formulation of the person’s interests when she was fully capable.

Nevertheless, we reject this approach on the grounds that dementia ought to be, can, and, importantly, *is* being rethought across a variety of discursive spaces. As we have shown, this framing of dementia is problematic since it overlooks an important set of dementia experiences. It is misleading and potentially exacerbates susceptibility to harm, stigmatization, and injustice. Scholars, advocacy groups, and health professionals alike have raised concerns about how people with dementia are treated and advocated for a reconsideration of this framing. Stressing that different models need not be mutually exclusive, we have laid down three possible ways of seeing dementia. First, considering dementia as a part of normal aging, i.e., a form of cognitive decline experienced by most as we grow old. On the other hand, PCC models, rather than focusing on what is lost, stress the importance of care and aim at preserving and enhancing personhood. Finally, more recently, embodied perspectives oppose reductionist accounts equating personhood to the brain by emphasizing that selfhood is also and necessarily embodied. There is a body memory in which one’s life experiences are sedimented and persistent, even in the case of dementia. While these different framings themselves are held up to critical scrutiny, they show that rethinking and reframing dementia is a distinct and fruitful endeavor.

Despite these available alternatives, advance directives remain anchored in a framework fostering different forms of epistemic injustice towards people with dementia. Considering these different forms of epistemic injustice (contributory, testimonial, hermeneutical), we advance that the entanglements between the unilateral framing of dementia and epistemic injustice raise several questions for the received view on advance directives.

For one, given the co-occurrence and the mutual reinforcement of communicative barriers and prejudicial dismissals of people with dementia’s testimonies due to testimonial and hermeneutical injustice, we can question whether caregivers and healthcare professionals are currently sufficiently equipped to tackle and inquire into the interests of people living with dementia. If – as empirical evidence suggests – our communicative sensibilities are, in general, unattuned to the various alternative ways those living with dementia might express their interests and informed by persistent biases on the epistemic capabilities of those living with dementia, testimonial injustices might lead to a (pre-emptive) dismissal or hampered understanding of the wishes and interests of those living with dementia. Moreover, the overemphasis on advance directives’ moral authority may amount to testimonial injustice. If, as Dworkin suggests, we take the interests expressed by people with dementia to be only experiential in nature (as opposed to critical), this may itself involve a preemptive and prejudicial failure to take seriously people with dementia as knowers and further inspire minimal engagement with those interests. The one-sided emphasis on the moral authority of advance directives may itself function to precisely confirm the credibility excess of clinical reports, health care professionals’ assessment, and legal documents over the testimonies of those living with dementia and their caregivers.

Second, public (and often publicized) debates on advance directives and euthanasia in the context of dementia, as important instances of meaning-making, may constitute ‘contributory injustice.’ These discussions, when uninformed or preceded by a more thorough exploration of dementia, populate and reinforce an existing social understanding of dementia and inform the conditions under which people draft advance directives. In this sense, those involved (and often in positions of power or affecting policy) fail to perform their epistemic due diligence by neglecting extant resources and, in effect, build upon a narrow and ultimately flawed understanding of dementia. Importantly, these remarks in the specific context of dementia ethics echo longstanding concerns expressed in feminist bioethics with regard to the detached (and therefore partial) view dominant in principlist bioethics. Bioethical scholarship – notably that of dementia ethics - fails to engage with its own situatedness in that many of its ‘arguments’ are based on intuitions uninformed by a wide body of literature and experiences of those most critically affected by those debates. We advocate for wider engagement with the experiences and narratives of those engaging in life writing, academic dementia studies, and activist accounts of dementia experiences.

Finally, given that social imagination is colored by a flawed understanding of dementia, we can question whether those drafting advance directives can be considered appropriately informed. In the decision to write up specific advance directives, one draws on one’s understanding and appreciation of one’s future condition. If those views are dominated by the monolithic understanding of dementia expressed above, it is likely that (potential) people with dementia’s decisions are informed by a particular framing of dementia.^18^ The fact that something akin to the disability paradox is widely reported for dementia, and there is evidence that people with early-stage AD push back or alter their advance directives suggests that a new outlook on dementia – informed by experience – alters their appreciation of the condition and their assessment of well-being in the context of dementia ^19^.

## Conclusion

In this paper, we suggested that the dominant framing of dementia as a ‘monster’ or a ‘destructive experience’ in clinical settings and lay audiences, in addition to stimulating bias and stigmatization towards people with dementia, similarly bears significantly on discussions of advance directives. The importance of the principle of autonomy anchoring the moral authority of advance directives and encouraging a distinction between the ‘then self’ and ‘now self’ suffering is, in important ways, indebted to a monolithic representation of dementia insensitive to the alternative epistemic resources arising from the experiences of people with dementia and scholarly engagement in dementia studies.

While the lack of engagement with such resources in dominant dementia meaning-making practices is problematic in and of itself, it raises three particularly thorny issues for the case of advance directives. First, the prejudicial dismissals of people with dementia’s testimonies due to testimonial and hermeneutical injustice led us to question whether healthcare professionals (and even relatives) are sufficiently ‘equipped’ to take into consideration the wishes and interests of the ‘now self,’ the person with dementia. Second, debates on advance directives may themselves constitute a case of contributory injustice when they are uninformed and reinforce an understanding of dementia crystallized in our social imaginary, which in return also informs the conditions under which people draft advance directives. Finally, given that a flawed understanding of dementia colors the social imaginary, we can question whether those drafting advance directives can be considered appropriately informed.

To reiterate, the problem we perceive with advance directives does not lie in the tool itself but in the framing in which it is rooted and operationalized. Rather than discarding the very idea of advance directives and their use, we do emphasize their importance as tools to be used carefully in the context of dementia. In other words, advance directives should not be taken as measures stipulating exactly what must be done when the moment has come, but rather as instruments in need of interpretation symbolizing the critical interests and view of a good life of the person with dementia. We also put forward that this should be done with a reappraisal of the ‘now self,’ a self that still has meaningful experiences and interests and is not just the leftovers of a ‘then self’ deprived of autonomy. In considering advance directives, the ‘now self’ carries a heavy weight, and maybe a heavier one than the ‘then self.’

## Data availability statement

The original contributions presented in the study are included in the article/supplementary material. Further inquiries can be directed to the corresponding author.

## Author contributions

FV: Writing – original draft, Writing – review & editing. DK: Writing – original draft, Writing – review & editing.

## Footnotes

1 We should note here that the legislation and implementation of advance directives can vary greatly from one country to another. For instance, in 2021, only 15 countries in the European Union had developed specific legislation on advance directives (10).

2 While we focus on the dominant view portrayed by Dworkin, we acknowledge other positions exist. Rebecca Dresser for instance has argued, against Dworkin, that the tenuous nature of advance directives fails to accurately capture how people’s interests evolve over time (Choi, 2022).

3 While the concept of harm can be used nonnormatively (X’s actions have adverse effects on Y), in the rest of the paper we will use harm normatively (if X harms Y, X also wrongs Y).

4 The principle of non-maleficence entails that one ought to refrain from harming persons. The principle of beneficence on the other hand is more demanding and goes beyond the one of non-maleficence as it requires that someone actively takes positive steps to contribute to the welfare of others.

5 In the 1960s’ with the development of neurological research, AD was used as powerful articulation of concerns about ageing. Robert N. Butler, a leading gerontological physician, urged for instance to replace the ageist image of senility with AD, specifically with the purpose to obtain public funding on the ground that AD was separable from normal ageing processes (Katz & Leibing, 2023).

6 As a recent review points out so-called ‘Dementia Friendly Initiatives’, combining insights from person-centered care and activist attempts to reframe dementia (cfr. later), are (increasingly) finding their way into care contexts promoting more inclusive, communal, and participatory dementia care models (14). Our concerns expressed here are, therefore, primarily addressed toward a lack of engagement with such alternative conceptions of dementia (care) in broader public and medical discourse. The failure of uptake of these initiatives expresses precisely the contributory injustice we discuss in section 3. We thank an anonymous reviewer for stressing this point.

7 Memory, especially, is critical, as it allows a person to make sense of her life and shapes her interest. Not only does memory ground autonomy, it also allows one to exercise this autonomy.

8 We need to stress here that if Kant was influential in the rise of autonomy as a central principle for biomedical ethics, his view was more stringent. For Kant, an agent has ‘autonomy of the will’ if and only if she acts in accordance with the categorical imperative. In biomedical ethics, autonomy, broadly construed, refers to that which makes judgments and actions one’s own. Autonomy then requires a capacity for intentional action and an independence from controlling influences (Beauchamp & Childress, 2019).

9 Mini Mental State Examination commonly used in these situations evaluates orientation, registration, attention and calculation, recall, and language.

10 For an extended discussion of identity and dementia see (19–21).

11 See discussion on advance directives in the previous section and, in particular, Dworkin’s position.

12 For exceptions, see footnote 6.

13 By no means do we intend to undermine or underestimate the tragicness and seriousness dementia may encapsulate, nor the value of a biomedical framework. Receiving a diagnosis and living with dementia is generally challenging, both for the person and her surroundings. Nevertheless, as we argue further having a more nuanced understanding of what living with dementia entails may benefit patients, families, and care givers alike.

14 This point is critical as it could also lead to another injustice by overlooking the fact that the environment may have a predominant role in the willingness of the ‘Now-Self’ to end their life or not. A person with dementia living in a flourishing environment with appropriate care may well rethink her advance directives, while one living in a poor care structure may, on the contrary, may be more inclined to follow them through.

15 Epistemic resources entail the concepts, language, and normative criteria we have available to make sense of our world and experiences (39).

16 Note than that these issues do not merely arise in cases of ‘missing or unsupported’ diagnoses. Rather, conditions such as endometriosis have been adequately described, and clear diagnostic conditions are stipulated. The widely reported issues in arriving at a diagnosis relate to a lack of understanding of the condition within the biomedical community. As such, even if ‘endometriosis’ or similar conditions are known, individual physicians may lack the necessary resources to properly understand their breadth and extent in actual patients.

17 See also work on dementia as transformative experience for similar arguments (Boerstler, 2017; Walsh, 2020).

18 See for example Cuadrado et al. (51). Here the authors show that presenting alternative framings using different representations of people with dementia led to a positive change in attitudes towards dementia. They contend that using such tools to develop policies targeting at communication of dementia can reduce the stigma associated with it.

19 While substantial suggestions to this end are beyond the scope of the present text, the alternative framings of dementia as they arise from activism or academic engagements in critical dementia studies can also positively inform advance care planning. In a recent interview study on advance care planning with people living with dementia and their carers, Phenwan et al. (52) stress the co-construction of the needs and decisions of people living with dementia and their carers to initiate and revise advance directives. As such, they echo the importance of considering the relationality of dementia care and advance care planning of (critical) dementia studies against the strict moral authority of advance directives. Another way to conceive of advance directives from the purview of alternative, more nuanced understandings was discussed by Widdershoven and Berghmans (2005). They suggest that rather than being objective decisive statements about prior wishes, advance directives are hermeneutic tools for joint meaning-making. As such, they are not statements to follow when time has come, but tools to orient shared practices of care capable of furthering the autonomy of the person.
